# Are hyoliths Palaeozoic lophophorates?

**DOI:** 10.1093/nsr/nwz161

**Published:** 2019-10-25

**Authors:** Fan Liu, Christian B Skovsted, Timothy P Topper, Zhifei Zhang, Degan Shu

**Affiliations:** 1 State Key Laboratory of Continental Dynamics, Shaanxi Key Laboratory of Early Life and Environments, Department of Geology, Northwest University, Xi'an 710069, China; 2 Department of Palaeobiology, Swedish Museum of Natural History, Stockholm SE-104 05, Sweden

**Keywords:** orthothecid, the Chengjiang Lagerstätte, Cambrian, soft parts, lophophore

## Abstract

The phylogenetic position of hyoliths has long been unsettled, with recent discoveries of a tentaculate feeding apparatus (‘lophophore’) and fleshy apical extensions from the shell (‘pedicle’) suggesting a lophophorate affinity. Here, we describe the first soft parts associated with the feeding apparatus of an orthothecid hyolith, *Triplicatella opimus* from the Chengjiang biota of South China. The tuft-like arrangement of the tentacles of *T. opimus* differs from that of hyolithids, suggesting they collected food directly from the substrate. A reassessment of the feeding organ in hyolithids indicates that it does not represent a lophophore and our analysis of the apical structures associated with some orthothecids show that these represent crushed portions of the shell and are not comparable to the brachiopod pedicle. The new information suggests that hyoliths are more likely to be basal members of the lophotrochozoans rather than lophophorates closely linked with the Phylum Brachiopoda.

## Introduction

Hyoliths are extinct invertebrates with calcareous shells that were common constituents of the Cambrian fauna and formed a minor component of benthic faunas throughout the Palaeozoic until their demise in the end-Permian mass extinction [1]. The skeletons of hyoliths are composed of a deep cone-shaped conch and a lid-like operculum, sometimes complemented by laterally projecting spines (helens) inserted between the conch and operculum [2]. Two distinct groups of hyoliths have traditionally been recognized: the orders Hyolithida and Orthothecida. Hyolithid conchs usually have a sub-triangular cross-section with an arched ventral extension of the aperture (ligula). The operculum of hyolithids is divided into distinct cardinal and conical shields by a prominent fold and its internal surface bears two sets of processes (cardinal processes and clavicles). Most hyolithids are also characterized by the presence of helens. Orthothecids have a variable (circular, quadrate, triangular, etc.) conch cross-section with a simple aperture (without ligula) and a flat, retractable operculum, often with cardinal processes but generally without distinct clavicles. No evidence exists to suggest that orthothecids had helens.

The biological affinity of hyoliths has long been controversial and the group has been compared with a number of animal phyla, most commonly the Mollusca or the Sipuncula, although other researchers have considered hyoliths as a separate ‘extinct phylum’ (see review in [1]). Recent discoveries of hyolith morphology have revealed a wealth of new data relevant for palaeobiological interpretations of hyoliths and their biological affinity. This includes detailing complex patterns of muscle scars in conchs, opercula and helens [2–5], as well as information surrounding the morphology, insertion and mode of formation of hyolithid helens [4,5]. However, information from the soft parts associated with the hyolithid operculum, including a tentaculate feeding organ [6,7], have promoted a new view of the hyolith body plan and phylogenetic affinity, and have been used to argue for a close link with lophophorates [7] or even for an interpretation of hyoliths as derived brachiopods [8]. Apparent support for this interpretation emerged recently from the interpretation of apical structures in an orthothecid from the Chengjiang Lagerstätte in South China, purportedly representing a soft brachiopod-like pedicle [9].

Here, we describe the first credible soft parts other than the alimentary canal of any orthothecid hyolith, *Triplicatella opimus* [10] from the Chengjiang biota of South China. The feeding apparatus of *T. opimus* differs from the previously described hyolithids and demonstrates a different mode of life for orthothecid hyoliths compared to their better-known relatives: the hyolithids. In a similar manner, investigation of new, extensive collections of orthothecid hyoliths with apparent apical ‘appendages’ from the Chengjiang and Shipai biotas provide strong evidence for a radical reinterpretation of the purported pedicle in these taxa, as remains of the crushed apical portion of the shell itself. The new data, together with detailed morphological and functional comparisons of the feeding organs of lophophorates as well as new data on hyolith shell structure [11–13], prompt a reassessment of the Hyolitha and their recently proposed phylogenetic position within the Lophophorata [7–9].

## *T. Opimus* from the Chengjiang biota and the nature of hyolith-feeding organs

**Figure 1.**
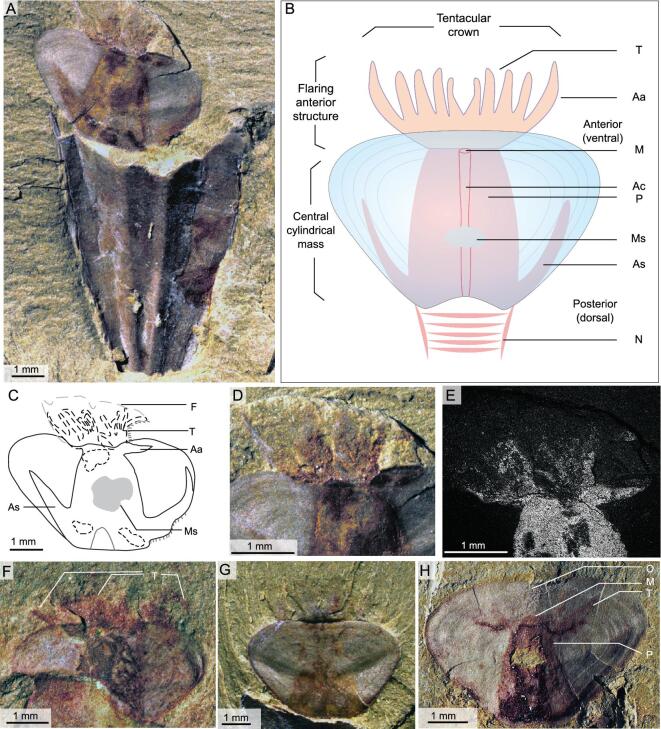
Soft tissues preserved in *Triplicatella opimus* from the lower Cambrian (Stage 3) Chengjiang Lagerstätte. (A) ELI H-113, conjoined conch and operculum with extended tuft-like tentacles. (B) Idealized reconstruction of the operculum of *T. opimus.* (C) Interpretative drawing of the operculum from (A). (D) Close-up view of (A), showing a crown of tentacles extending from the anterior margin of the operculum. (E) Backscatter electron micrograph of the operculum from (D) highlighting the individual tentacles. (F) ELI H-0011B, an operculum, showing the central mass and tentacles. (G) ELI H-120B, showing the impression of the tentacular organ outside the margin of the operculum. (H) ELI H-168A, showing the retracted tentacular organ inside the margin of the operculum. Aa, anterolateral arm; As, arcuate structure; Ac, alimentary canal; F, fan-shaped discolouration; M, mouth; Ms, muscle scar; N, neck; P, pharynx; T, tentacle.

**Figure 2.**
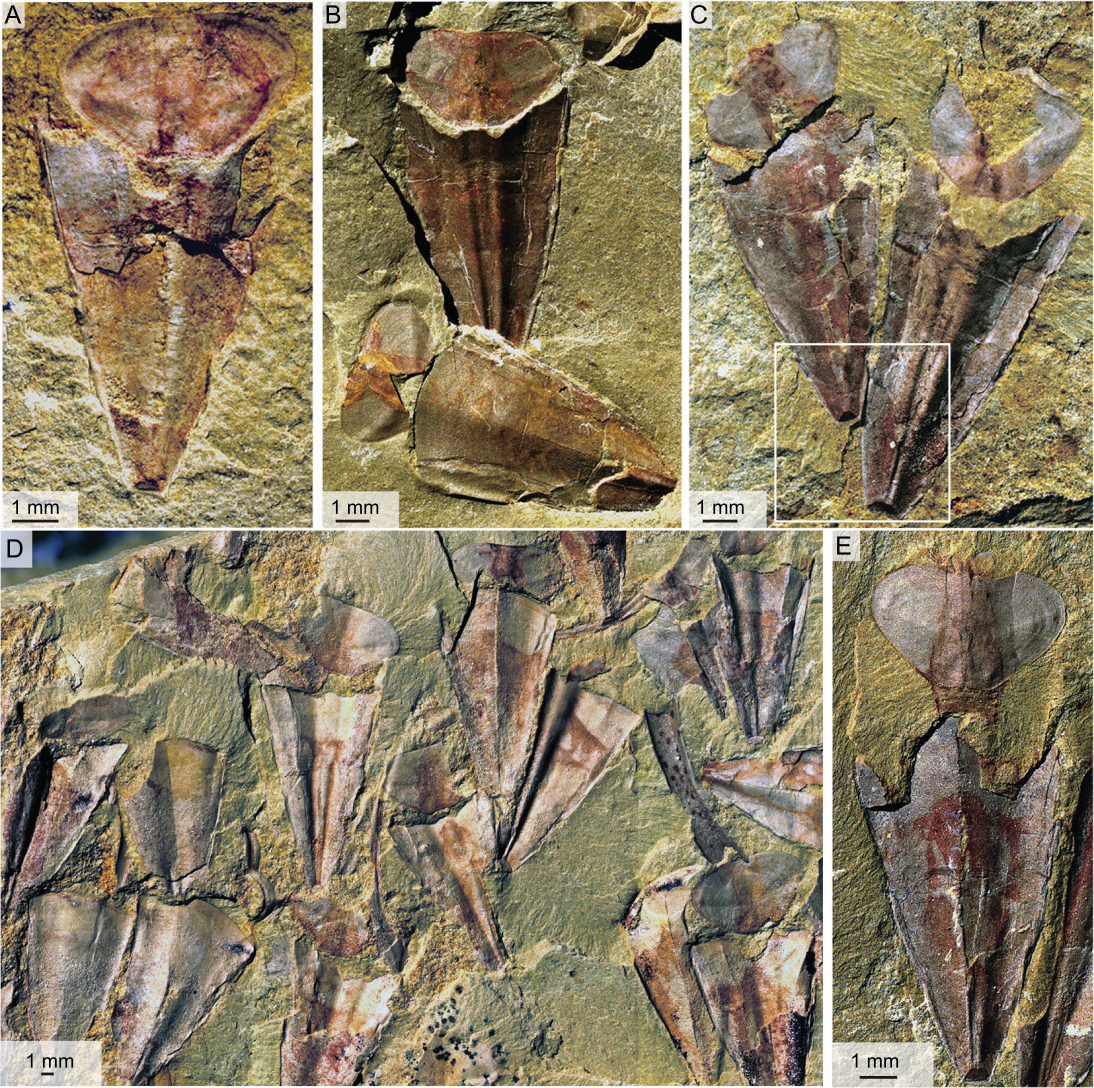
Soft parts associated with the opercula and conch in *Triplicatella opimus*. (A) ELI H-126A, conch with its operculum that has been slightly withdrawn inside the aperture of the conch. (B) ELI H-188A, two specimens showing imprints of the cylindrical mass on the interior of the opercula. (C) ELI H-126A, two conjoined individuals with reddish-brown tinges on the dorsal interior surfaces, showing the trapezoid-shaped apex marked by a box. (D) ELI H-183, populous occurrence of *T. opimus* on a slab. (E) ELI H-176A, specimen showing the neck-like band connecting the dorsal margin of the operculum and the internal region of the apertural conch.

**Figure 3.**
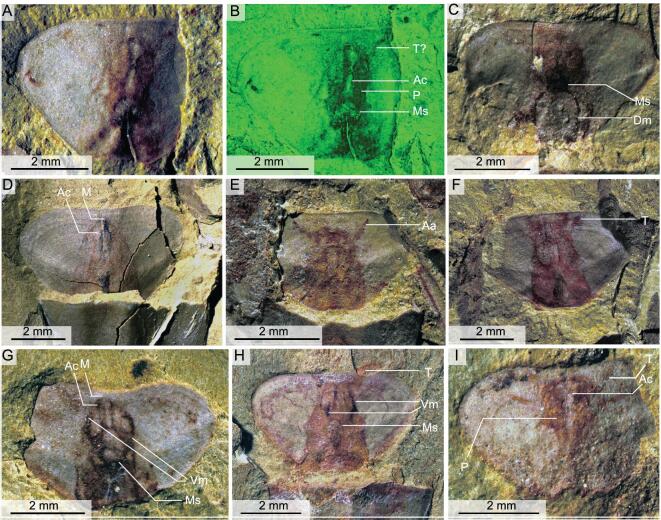
Soft-tissue imprints on the opercula of *Triplicatella opimus* from the lower Cambrian (Stage 3) Chengjiang Lagerstätte. (A) ELI H-176B, a sub-triangular-shaped operculum, with imprints showing the central mass structure. (B) Fluorescence microscope analysis of soft tissues in (A). (C) ELI H-191B, showing the muscle scar stained with a reddish-brown tinge and the impression of dorsal muscle scars. (D) ELI H-152, operculum with the pharynx and mouth. (E) ELI H-183B, note the two recognizable retracted tentacles. (F) ELI H-127A, showing red tinges of the retracted tentacles. (G) ELI H-125A, operculum showing the central mass structure with imprints of ventral muscle stars. (H) ELI H-115A, specimen showing retracted tentacles and muscle scars. (I) ELI H-170A, specimen showing the retracted feeding organs with six distinguishable tentacles anterior to the cylindrical mass. Aa, anterolateral arm; Ac, alimentary canal; Dm, posterior (dorsal) muscle scar; M, mouth; Ms, muscle scar; P, pharynx; T, tentacle; Vm, anterior (ventral) muscle scar.

A large number of specimens of a hyolith preserving imprints of the soft anatomy have been recovered from the Chengjiang biota of South China (see Supplementary material). This species, which exhibits a rapidly expanding triangular shell, was most recently informally attributed to the hyolithid taxon *Linevitus opimus* [10,14]. While this species is superficially similar to hyolithid hyoliths in having a conch with a sub-triangular cross-section (Figs 1A and 2), it lacks helens and a differentiated ligula on the ventral aperture of the conch (Figs 1 and 2). Associated opercula lack both cardinal processes and clavicles (Figs 1–3). This species should consequently be excluded from the Hyolithida. We consider this taxon to be most closely comparable to the orthothecid genus *Triplicatella* (see Supplementary material), previously known mainly from disarticulated opercula in Small Shelly Fossil (SSF) assemblages [15]. Although both conch and operculum are sometimes found in isolation, many specimens preserve the operculum associated with the conch (Figs 1A and 2) and it may be found either partly inside or just in front of the conch aperture. In all such specimens, the operculum is slightly displaced from the conch and, in most cases, they are preserved as internal moulds. The opercula are rotated to a nearly horizontal position, invariably with the dorsal side closest to the conch, indicating that the main attachment of the operculum to the conch was along this part of the margin.

**Figure 4.**
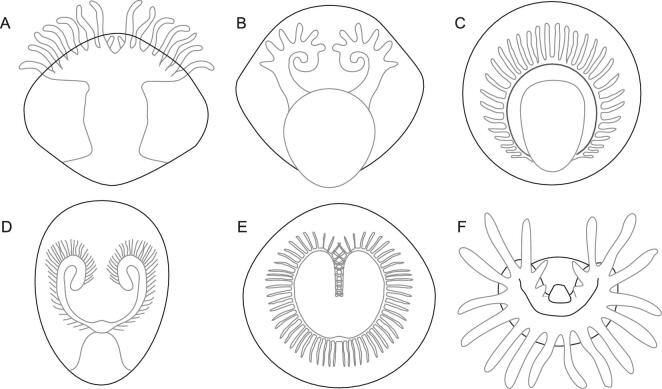
Interpretative line drawings of lophophore organization in hyoliths, Cambrian and extant lophophorate taxa. (A) The hyolithid *Haplophrentis*, modified from [7]. (B) The stem lophophorate *Yuganotheca elegans*, modified from [26]. (C) The Cambrian brachiopod *Heliomedusa orienta*, modified from [25]. (D) The linguloid brachiopod *Lingulellotreta malongensis*, modified from [62]. (E) The schizolophe lophophore of the living brachiopod *Pumilus antiquatus*, modified from [18]. (F) The extant phoronid, *Phoronis ovalis*, modified from [21].

### Soft parts associated with the opercula of *T. opimus* and its feeding organs

A number of opercula of *T. opimus* preserve imprints of soft parts in the form of red-, yellow- or brown-stained patches (Figs 1–3). These patches are most strongly developed directly under the operculum itself but may extend beyond its margins. Three distinct structures may be preserved: (i) a flaring anterior structure (Fig. 1A–F); (ii) a central, cylindrical mass (Figs 1A–C, H, 2B, C, E and 3); and (iii) arcuate structures that follow the lateral margins of the operculum (Fig. 1A–C). The flaring anterior structure is preserved in a shallow, fan-shaped arrangement, with the entire structure extending approximately 2.5 mm in width and 1–1.5 mm in length. The flaring anterior structure is composed of one pair of straight or gently bending anterolaterally directed bands or arms that may project beyond the margin of the operculum (Fig. 1A–F). Between these two anterolateral arms (Fig. 1B) lies a poorly defined crown of radiating, tentacle-like structures emanating in a tuft-like manner from the anterior margin of the arms (Fig. 1A–E). In the best-preserved specimen, individual, elongate tentacles can be recognized as pyrite-replaced members (Fig. 1D and E) and the tentacles appear to be particularly prevalent in the central region of the two arms (Fig. 1D and E). However, for the majority of specimens, individual tentacles are not discernible (Fig. 1F and G); instead, the flaring anterior structure is commonly preserved as simply a fan-shaped impression or a faint discolouration on the rock surface that fringes the anterior margin of the operculum (Fig. 1A and C–F). Although there are strong indications that this extending anterior structure represents a tentacular feeding organ and we herein consider it as such, the quality of preservation has hindered our understanding of the precise number and arrangement of tentacles within the structure (Fig. 1G). In some specimens, the anterolateral bands and tentacular crown is retracted back to the dorsal margin of the operculum (Figs 1H, 3E and I) and the tentacles do not project beyond the ventral margin (compare Fig. 1A and H).

The flaring anterior structure is medially connected at the base to a central cylindrical mass. This central mass consists of a cylindrical structure that tapers from the posterior (dorsal) end of the operculum towards the anterior (ventral) (Figs 1, 2B–E and 3D–H) and is interpreted here as the pharynx and associated muscles that connect the structure to the operculum and to the neck of the organism (Figs 1B and 2E). Although the outline of the central cylindrical mass is regular, the internal structure of the central mass is variable. Medially positioned dark-stained or light areas, towards the anterior of the central mass, are present in some specimens (Figs 1A, H and 3A–H). The position of these structures in relation to the tentacular feeding organ has led us to interpret them as representing an anterior mouth together with the proximal portion of the alimentary canal (Fig. 3A–H). The anterior part of the central mass contains a central, light-coloured area that represents the continuation of the alimentary canal from the base of the food-collecting organ (Fig. 3A, B, G and I). Dark-coloured areas lateral to this canal may represent anterior (ventral) muscles (Fig. 3G and H) that provide attachment to the operculum. It is very likely that the pharynx was attached to the operculum because the pharynx and tentaculate structure remain in the same central position of the operculum, even when the operculum is isolated, having been displaced from the conch (Fig. 3A, B, G and I). Posteriorly, the central mass continues outside the operculum in the form of a dark-stained neck towards the dorsal margin of the aperture of the conch (Figs 2B, C, E and 3E–H). The neck is interpreted here to represent the connection of the soft parts associated with the opercula with the rest of the organism in the conch (Fig. 2E). The posterior position of the neck explains the rotation of the operculum with the posterior end towards the conch aperture.

The lateral arcuate structures are attached to the posterior of the central mass and appear to follow the margins of the operculum (Fig. 1A). These structures are variable in morphology and position, but are generally best expressed along the posterolateral margins and they often taper and fade towards the anterolateral ends of the operculum. However, in other specimens, the arcuate structures appear to follow the growth lines of the operculum and they may sometimes encircle the operculum completely (Fig. 2A). As they appear to follow the growth lines of the operculum, we interpret these structures to represent the margin of shell-secreting epithelia responsible for its formation.

### *Triplicatella* and *Haplophrentis*

Well-preserved specimens of the hyolith *T. opimus* with associated conch and operculum from the Chengjiang Lagerstätte yields the first information on the soft anatomy, with the exception of the gut, of orthothecid hyoliths (Figs 1–3). The soft parts associated with the operculum of *Triplicatella* do show similarities in gross morphology with those described from the hyolithid *Haplophrentis* from the middle Cambrian Burgess and Spence shales of North America [7]. The soft tissues in both taxa display a central body that flares ventrally into a tentaculate organ. However, there are also a number of distinct morphological differences between the two genera.

The central mass associated with the operculum of *Triplicatella* is similar to the centrally located ‘pharynx’ of *Haplophrentis*. Both masses are tubular to cylindrical in shape and house a central alimentary canal or pharynx lumen [7]. The main differences between the pharynx and central mass of the two taxa appear to be its flexibility and the ability of the organ to extend and retract. Variations in morphology were used as evidence to indicate that the pharyngeal organ of *Haplophrentis* was protrusible [7]. In some specimens, the pharynx is tubular in shape and extends almost to the anterior margin of the operculum, projecting the tentacles beyond the margin of the operculum (Figs. 1b and 2d in [7]), while, in other specimens, the pharyngeal organ is retracted, preserved as an indistinct circular structure towards the anterior of the operculum, with the distal tentacles entirely concealed beneath the operculum (Fig. 2a-c in [7]). The central mass of *Triplicatella* does not display such morphological variation and instead is relatively consistent in shape, suggesting that, comparatively speaking, it was a more rigid structure compared to the pharynx of *Haplophrentis*. That is not to say that *Triplicatella* did not possess the ability to retract its feeding organ within its shell, as the position of the feeding organ is variable among *Triplicatella* specimens and the structure may be either retracted beneath the operculum (Figs 1H, 3E and I) or extend beyond its anterior margin (Fig. 1A, D, F and G). However, in both cases, the central mass does not change shape and remains preserved as a tapering cylindrical structure. This suggests that the central mass did not itself contract (as in *Haplophrentis*), but rather it is most likely that muscles associated with the neck structure of *Triplicatella* (Fig. 2E) controlled the movement of the feeding organ. With the central mass attached to the operculum, such actions would have also controlled the movement of the operculum relative to the conch. *Haplophrentis* does not obviously possess a comparable structure to the neck of *Triplicatella*, with the pharynx of *Haplophrentis* directly joining the gut under the posterior margin of the operculum (Fig. 1b in [7]). The possession of a neck may not have been necessary, as the pharynx of *Haplophrentis* controlled the movement of the feeding organ and as the operculum was permanently situated at the aperture and not withdrawable inside the conch as in *Triplicatella*. The arcuate structures that we have interpreted as lateral shell-secreting epithelia in *Triplicatella* also lack a counterpart in preserved specimens of *Haplophrentis*.

Despite their poor preservation, the anterior flaring structures of *Triplicatella* broadly resemble the feeding organ identified in *Haplophrentis*, but also appear to differ in some important ways. For instance, the anterolaterally directed arms are clearly homologous with the ‘gullwing-shaped band’ in *Haplophrentis* [7]. However, the anterolateral arms in *Triplicatella* are well defined, regardless of whether the organ has been retracted or extended, unlike the gullwing-shaped band in *Haplophrentis*, which becomes indistinguishable from the pharynx when the feeding organ has been retracted within its shell (Fig. 2a-c in [7]). The tentaculate structure in *Triplicatella* also appears noticeably different, with the tentacles seemingly attached in relatively dense clusters on the central, anterior margin of the anterolateral arms, instead of 12–16 individual elements being evenly distributed along the length of the band as in *Haplophrentis* (Figs. 1 and 2 in [7]). The morphological differences in the arrangement of the tentaculate organ further suggest that the function of the feeding organ differed between orthothecid and hyolithid hyoliths.

## Is it a lophophore?

Hyoliths have recently been suggested to have found a home within the lophophorates, based on the discovery of *Haplophrentis* specimens with an extendable, tentacle-bearing feeding organ [7]. Despite this phylogenetic placement hinging on the interpretation of this tentaculate structure as a lophophore, a comparative study was sorely lacking. A lophophore is a complex feeding organ present in all members of the Lophophorata [16] and is defined as a row of ciliated tentacles that surround the mouth and filter particles from the water currents created by the cilia [17]. Tentacles in lophophores tend to be elongated, evenly distributed and densely arranged, forming a fence-like row to effectively filter food particles by the creation of a feeding current through coordinated movements [17–19].

The feeding organ of *Haplophrentis* displays a relatively simple arrangement of 12–16 elongated, tapering tentacles (Fig. 4A) that flank a centrally located mouth [7]. The cilia, if originally present, are not preserved [7]. This simple arrangement was noted by the authors [7] to be distinct from the complex lophophore arrangements of adult members of the Lophophorata and instead the gross morphological similarities between the lophophore of a larval brachiopod (*Glottidia*) and the tentacular organ of *Haplophrentis* were used to provide support for a lophophore interpretation and a benthic suspension-feeding life habit [7]. However, the similarities in general morphology is somewhat misleading, as lingulid brachiopod larvae (such as *Glottidia*), in addition to feeding, primarily use their lophophore for swimming in the water column and the structure of the lophophore changes soon after settlement and metamorphosis, when the ability to swim is no longer necessary [18–20].

Despite the structure of the lophophore in members of the Lophophorata being highly variable [21–23] and susceptible to environmental conditions [24], it is invariably a curved structure that encircles the mouth (Fig. 4B–F). The lateral disposition of the tentacles in *Haplophrentis* with a centrally located mouth (Fig. 4A) bears little resemblance to the shape of even the simplest ringed trocholophe or schizolophe lophophore of extant brachiopods (Fig. 4E) or the ovoid lophophores of modern phoronids (Fig. 4F) [18,19].

The differences with the lophophore of extant taxa were noted by Moysuik *et al.* [7] and instead similarities were highlighted between *Haplophrentis* [7] and early Cambrian brachiopods, such as the lophophore of the enigmatic *Heliomedusa* [25]. However, the two structures display notable differences (compare Fig. 4A and C). The lophophore of *Heliomedusa* is characterized by two curved brachial axes that surround a central mouth, with each arm bearing a row of long, slender, closely spaced tentacles (Fig. 4C) [25]. The tentacles are hollow, bearing a tentacular canal, and are distinctly ciliated, as are the brachial axes, presumably to facilitate transport of particles to the centralized mouth [25]. The presence of hollow, ciliated tentacles that surround a central mouth provides unequivocal evidence for a lophophore in the early Cambrian (first appearance in Stage 3) brachiopod *Heliomedusa* [25].

These distinctive features cannot be observed in the feeding organ of *Haplophrentis* (Fig. 4A) and their absence places doubt over the interpretation that the feeding structure in *Haplophrentis* represents a lophophore (as observed in crown-group lophophorate taxa). As in *Haplophrentis*, these morphological features cannot be observed in the tentaculate feeding organ of *Triplicatella*. Although the preservation of the individual tentaculate elements hinders a detailed comparison, the tuft-like, clustered configuration of tentacles does not resemble any lophophore arrangement displayed in crown-group lophophorate taxa. The anterolateral arms, although flanking a central mouth, exhibit a lateral arrangement, are never strongly curved and do not appear to encircle the interpreted location of the mouth (Fig. 1B). The lack of ciliated tentacles could simply be attributed to preservational loss, although, together with the morphological differences described above, there is limited evidence to suggest that the structure resembles a lophophore of a crown-group lophophorate.

Some general morphological similarities can be made with the feeding apparatus of the stem-group lophophorate (recently reinterpreted as a stem-group brachiopod [9]), *Yuganotheca elegans* (Fig. 4B) [26]. Both *Haplophrentis* and *Yuganotheca* possess a relatively thick tentaculate apparatus that is not obviously ciliated, although the organ in *Y*. *elegans* is horseshoe-shaped and the tentacles hollow—much more comparable to the lophophore of phoronids and brachiopods. In light of this, the feeding apparatus of *Yuganotheca* is most likely homologous with the lophophore [26]. However, without additional lophophore characteristics, the morphological similarities between the feeding structures of *Yuganotheca* and hyoliths may be superficial and there is not sufficient evidence to consider that the feeding organ of hyoliths is homologous to the lophophore seen in crown-group (or even stem-group) lophophorates.

This acknowledgement that the hyolith-feeding organ does not represent a lophophore, however, does not alter the suspension-feeding lifestyle proposed for *Haplophrentis* by Moysiuk *et al.* [7], as the general disposition of the feeding structure, consisting of a lateral band with relatively closely and equidistantly spaced projections, is a configuration commonly observed in filtering structures across the animal kingdom, such as the tentacles of sabellid and serpulid polychaetes [27] or the tentacles of pterobranchs [28]. This lateral arrangement of extensions would be proficient for the extraction of suspended particles from the water and the presence of this tentacular organ, together with the possession of helens to lift the aperture of the hyolith above the substrate to avoid fouling, provides convincing evidence that *Haplophrentis* (and perhaps by analogy other hyolithids as well) was a suspension feeder. Unlike *Haplophrentis*, which displays an evenly distributed row of tentacles along the gullwing-shaped band, *Triplicatella* instead possesses a clustered tuft of tentacles that appear to be more numerous towards the central region of the two arms (Fig. 1A–F). This configuration seems less than optimal for suspension feeding (for the reasons outlined above), suggesting that this taxon (and by analogy other orthothecids) may have had a different life mode.

## Orthothecid hyoliths with ‘pedicles’

Sun *et al.* [9] recently illustrated apical structures in the orthothecid hyolith *Pedunculotheca diania* Sun, Zhao & Zhu in [9] from the Chengjiang biota of South China that were interpreted as peduncular structures ending in a digitate holdfast. The apparent ‘pedicles’ were only briefly described, but the presence of this structure was used as an argument to support the placement of hyoliths in the stem group of the Brachiopoda (Fig. 4 in [9]), nesting between different tommotiid (such as *Paterimitra* and *Micrina*) and soft-bodied taxa (such as *Yuganotheca*). The interpretation that the apical structure in *P*. *diania* represents a pedicle is, however, at odds with a number of well-established morphological characteristics of hyoliths, in particular the ubiquitous closed apex of the hyolith conch (in both hyolithids and orthothecids), that exist from the earliest larval to adult ontogenetic stages [5,29]. This somewhat perplexing paradox has prompted an investigation of a large number of specimens of *P. diania* from the Chengjiang biota in addition to specimens of morphologically similar hyoliths from the Shipai Formation (see Supplementary material) in order to explore the validity of these claims.

### Apical structures in orthothecids from the Shipai Formation

**Figure 5.**
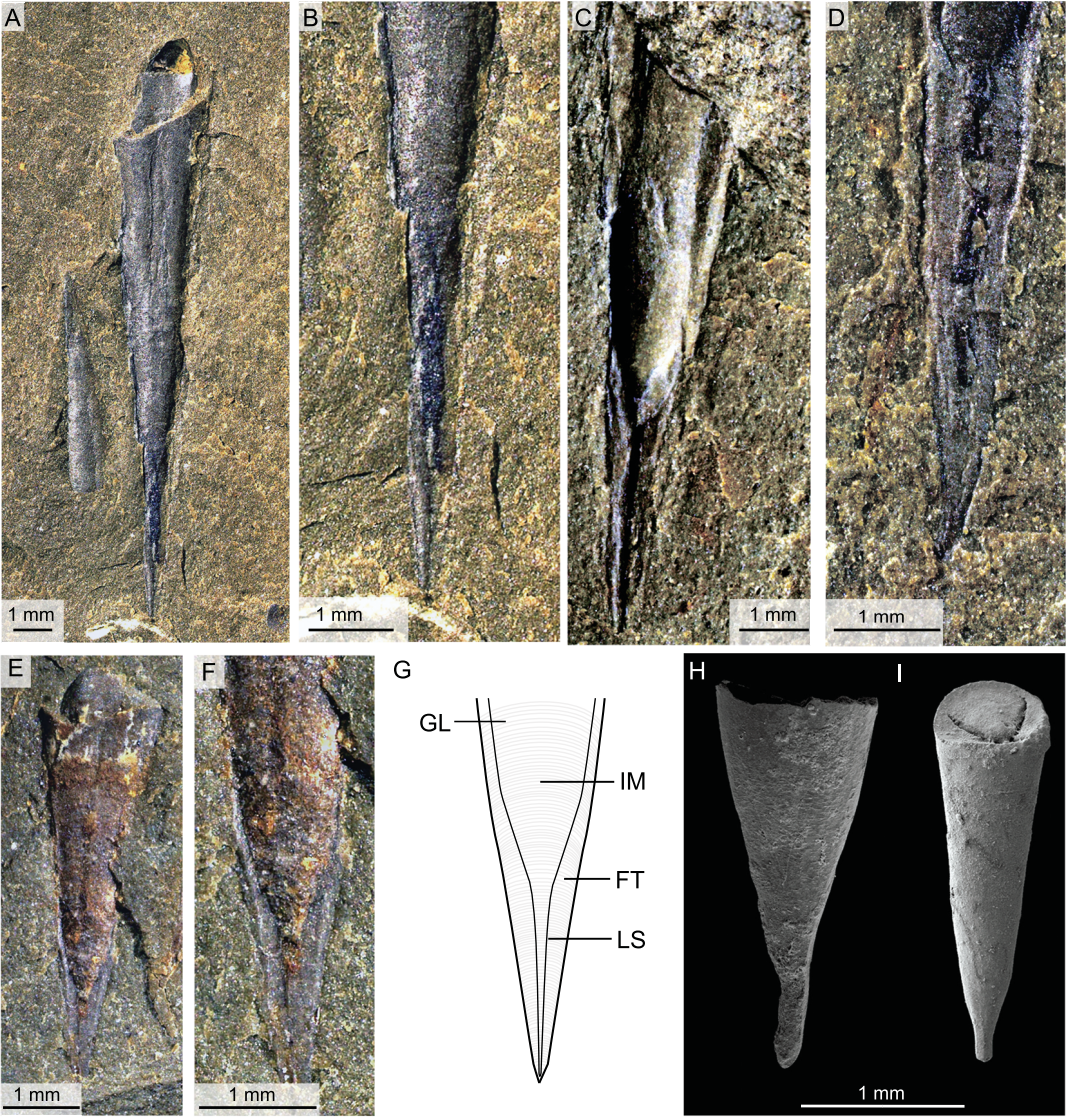
Apical structures in the orthothecid hyolith from the Shipai Formation in South China. (A) ELI QJP-SP-H-093, an individual showing longitudinal cracks through the shell but retaining slight topography due to partial sediment infill. (B) Close-up view of the apical part in (A). (C) ELI QJP-SP-H-202, interior of external mould of hyoliths from the Shipai Formation, showing the linear continuation towards the pointed conch apex. (D) ELI QJP-SP-H-143, close-up view of the apical part, displaying flattened triangular areas and a linear tube towards the pointed conch apex. (E) ELI QJP-SP-H-225, a flattened conch surrounding the central tube with the apical part of the shell in perfect continuation with the main part of the conch. (F) Close-up view of the apical part in (E). (G) Reconstruction of the apical part in the conch of *P. diania*. (H) and (I) ELI QJP-SP-H-SSF-8100031 and ELI QJP-SP-H-SSF-8104050, two small shelly fossils with a circular cross-section from the calcareous pelite layers in the Shipai Formation that predominantly represent internal moulds of the apical portion of the conch. FT, flattened triangular area; GL, growth line; IM, internal mould; LS, linear structure.

The Shipai Formation of the Three Gorges area [30] has yielded a number of hyolith specimens preserved in fine-grained shale and as phosphatized internal moulds (Fig. 5H and I) in two associated calcareous pelite layers. The shale-hosted specimens are slowly but regularly expanding conchs, sometimes associated with circular opercula (Fig. 5I), and are morphologically similar to *P. diania* from the Chengjiang biota. The shale-hosted specimens are flattened and show signs of brittle deformation in the form of longitudinal cracks through the shell but often retain slight topography due to partial sediment infill (Fig. 5A–F). In the apical portion of the conch, the sediment infill narrows more rapidly than the conch diameter itself and develops into a narrow, linear tube towards the pointed conch apex (Fig. 5B–G). The flattened shell surrounding the central tube is sometimes missing, but well-preserved specimens show that this part of the shell is in continuation with the main part of the conch, with a similar rate of expansion (average about 16.3°) and surface sculpture (Fig. 5B). Specimens from calcareous layers invariably have a circular cross-section and mainly represent internal moulds of the apical portion of the conch, replicating the unusual funnel-shaped apical constriction and the linear tube of the shale-hosted specimens, which shows that they most likely represent different states of preservation of the same taxon (Fig. 5H and I).

### Apical structures in *P. diania* from the Chengjiang biota

**Figure 6.**
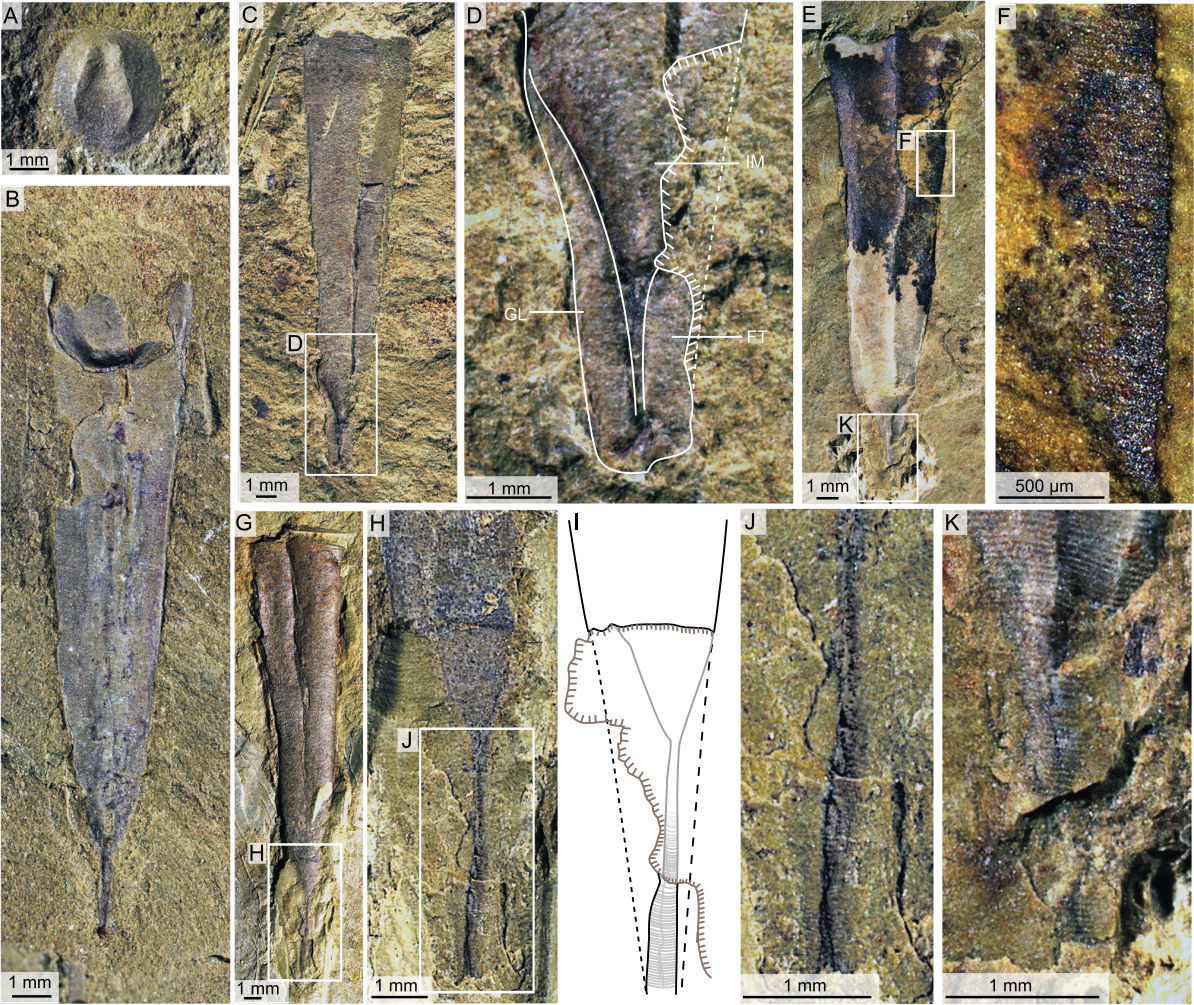
Apical structures in the orthothecid hyolith from the Chengjiang Biota. (A) ELI SK-0148A, interior of internal mould of operculum. (B) ELI SK-404A, a complete specimen of *Pedunculotheca diania* Sun, Zhao & Zhu articulated with operculum. (C) ELI SJZ-H-1348, the internal mould of the apical part of the conch in *P. diania* Sun, Zhao & Zhu, formed by sediment infill. Detail of marked box shown in (D) with outlines. (E) ELI SJZ-H-202, a conch with the incomplete apical structure. (F) Close-up view of the growth lines in the middle of the conch in (E). (G) ELI SJZ-H-1560, the specimen shows that the apical part of the conch in *P. diania* that typically displays a rapid decrease in diameter. (H) Close-up view of (G). (I) The interpretative drawing of (H). (J) Close-up view of (H) in box, showing that the linear continuation in the apical part of the conch of *P. diania* is ornamented by fine transverse lines. (K) Detail of the apical part in (E). FT, flattened triangular area; GL, growth line; IM, internal mould.

The conchs of *P. diania* from the Chengjiang biota are gently tapering cones (Fig. 6). All specimens are flattened, although many retain a slight topography due to partial sediment infill of the conch. Most specimens display longitudinal fractures testifying to the original rigidity of the conch. The specimens are often associated with circular opercula, indicating that the conch originally had a circular cross-section [9]. The original calcareous shell material has been demineralized during diagenesis, as is generally the case for trilobite sclerites and other calcareous skeletons in the Chengjiang biota [31]. The majority of conchs in our collection are moulds that generally preserve both the fine transverse growth lines of the external shell and the shape of the internal chamber of the conch as a result of sediment infill.

The internal mould of the apical part of the conch in *P. diania* typically displays a rapid decrease in diameter compared with the gradual expansion of the rest of the shell (Fig. 6B–E, G and H). This linear tube of the internal mould, in the apical region of the conch, is variable in length and typically shows slight topographic relief but it is always straight, and is flanked by flattened triangular areas (Fig. 6D; also see Fig. 2a and b in [9]). Well-preserved specimens show that the linear ornamentation of the conch (representing external ornamentation) continues uninterrupted across the thin, tubular internal mould into the surrounding flattened triangular areas (Fig. 6G–J) and we note that the lateral margins of the triangular areas form a continuation with the rest of the conch margins. Specimens in our collection where the linear, tubular structure has been partly removed show both positive and negative moulds with transverse ornament, testifying that the structure was hollow and partly filled by sediment (Fig. 6C and D).

### Is it a pedicle?

The linear apical structure and flattened triangular area as in specimens of *P. diania* were interpreted as the remains of an organic pedicle by Sun *et al.* (Fig. 1c and d [9]) with the median, linear structure interpreted as a coelomic cavity. The specimens from the Chengjiang biota available to us are identical to those illustrated by Sun *et al.* [9] in the general morphology of both conch and operculum, in surface ornament and even in the details of the apical structures (compare Fig. 6 to Figs 1 and 2 in [9]). Very little evidence was presented by Sun *et al.* [9] to justify their ‘pedicle’ claims and we here find no evidence to support their conclusion that the apical construction represents a peduncular attachment structure, but rather indicate that this area simply represents the apical region of the hyolith conch itself. The apical part of the orthothecid specimens from the Shipai Formation are morphologically identical to *P. diania* from the Chengjiang biota, with both collections displaying a rapidly tapering cone-shaped to linear 3D structure that is flanked by flattened triangular areas (Fig. 5A–F). However, the better preservation of the

lateral parts of the apex in specimens from Shipai allows a better understanding of how this part of the conch is preserved (Fig. 5G).

Our evidence is two-fold, as neither the general morphology nor the preservation of the apical structure supports the interpretation that this structure represents a pedicle. The lateral margins of the so-called ‘pedicles’, when preserved, are in continuation with the lateral margins of the main part of the hyolith conch (Figs 5 and 6), displaying a similar rate of expansion and even surface sculpture, with the posterior of the conch terminating in a pointed apex (Figs 5B and 6D). Brachiopod pedicles, although variable in morphology [32–34], are generally cylindrical and, despite occasionally tapering [34], seldom, if ever, taper to a pointed apex. The transverse striations on the apical structure, we presume, were one of the characteristics that persuaded Sun *et al.* [9] to consider this construction a pedicle. Brachiopod pedicles in the Chengjiang biota are frequently preserved with distinct annulations and well-defined contour lines [34] and their annulations are noticeably disparate from the ornament on the brachiopod shell [34]. The transverse striations on the apical structure of *P*. *diania* are identical to those on the conch—an observation initially recognized by Sun *et al.* [9], who commented that the striations on the ‘attachment stalk’ have an external ornament ‘whose spacing and relief is consistent with the ridges that ornament the conical shell’. This similarity in ornament is not coincidental, as specimens illustrated herein from Chengjiang and the Shipai Formation clearly show that the shell ornament is continuous across the conch, the lateral areas (labelled ‘venter’ in Fig. 1 in [9]) and into the linear structure (labelled as the coelomic cavity in Fig. 1 in [9]) (Fig. 6E, F and K). This observation of a continuous shell ornament across the conch and the previously interpreted ‘pedicle structure’ as defined by Sun *et al.* [9] in *P. diania* confirm that these apical structures are parts of the shell itself and not soft parts of a pedicle. The pedicle in at least one specimen apparently terminates in a digitate holdfast (Fig. 1a in [9]). This terminal holdfast is poorly defined and the only other illustrated specimen (Fig. 1c in [9]) that exhibits a questionable holdfast is not directly associated with the pedicle structure itself. We have found no comparable holdfasts in our material of *P*. *diania* from the Chengjiang biota and it is most likely that this lone example represents a chance juxtaposition of an unknown element at the apex of the hyolith conch.

**Figure 7.**
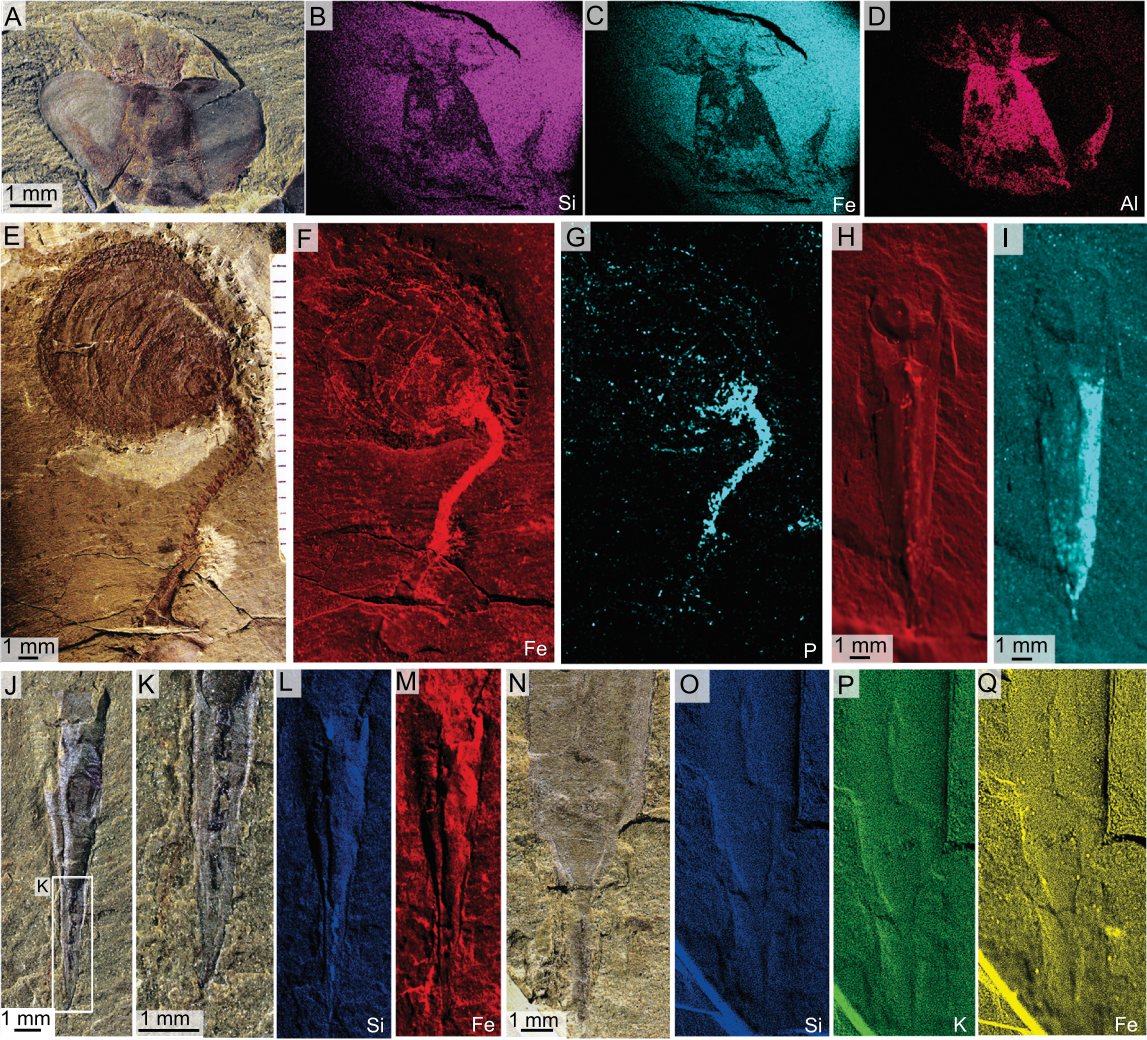
Elemental distribution in hyoliths and a brachiopod, from the Chengjiang biota and the Shipai Formation. (A) ELI H-113, the orthothecid *Triplicatella opimus.* (B)–(D) Elemental mapping of the opercula of *T. opimus*. Brighter colours represent higher concentrations of elements, showing elevated concentration of Fe on the soft feeding organ. The concentrations of Si and Al are decreased on the feeding organ, but are evenly distributed across the remainder of the specimen and the matrix. (E) The brachiopod *Obolella* with a preserved pedicle. (F) and (G) Elemental mapping of *Obolella*, showing elevated concentration of Fe and P on the pedicle. (H) and (I) ELI SK-404A, elemental distribution in the orthothecid hyolith *P. diania* from the Chengjiang biota. (J) ELI QJP-SP-H-143, the orthothecid hyolith from the Shipai Formation, displaying flattened triangular areas and a linear tube towards the pointed conch apex. (K)–(M) Elemental mapping of the (J), showing essentially no difference between the distribution of elements across the conch and the apical area. (N) ELI SJZ-H-1559, the orthothecid hyolith *Pedunculotheca diania* Sun, Zhao & Zhu. (O)–(Q) Si, K and Fe maps showing that essentially there is no difference between the distribution of elements across the conch and the apical area in (N).

Soft parts of animals in the Chengjiang biota are also generally preserved as iron oxides derived from pyrite grains [31]. This also applies to the pedicles (and other soft anatomy) of brachiopods (Fig. 7E–G) that are, when preserved, replaced by iron minerals, both in the Chengjiang biota [34] and in the taphonomically similar Guanshan biota [35]. However, the conchs and ‘pedicles’ of *P. diania* exhibit no noticeable iron content (Fig. 7H–Q), which is contradictory with the preservation of soft anatomy in other hyoliths in the Chengjiang biota, such as the tentaculate apparatus in *T. opimus* that does show a relative increase in iron (Fig. 7A–D). This dearth of iron contents in the apical structure of *P*. *diania* is in accordance with our interpretation that the Chengjiang specimens represent internal moulds.

In our interpretation, the apical part of the conchs of *P. diania*, like the orthothecid specimens from the Shipai Formation, are characterized by a cone-shaped apical cavity with a narrow central canal that continues towards the apex (Fig. 5G). Sediment infill of the conch and central canal provides a degree of three-dimensionality to the specimens, even after diagenetic decalcification and flattening of the shell. The apparent linear continuation (the ‘pedicle’ in [9]) of the conch in some specimens is a preservational artefact, as the flattened areas lateral to the central canal are vulnerable to fractures or may be covered by sediment (obvious in Fig. 6H–J). The transverse ornament of the ‘pedicle’ represents the external ornamentation of the shell, while the ‘coelomic cavity’ as interpreted by Sun *et al.* [9] represents the internal mould of the central cavity at the conch apex. The reason for the sharp decrease in diameter of the internal mould in the apical portion of the conch, however, is unclear. We note that many orthothecid hyoliths retain the ability to secrete shell material within the conch in the form of transverse internal septa and it is possible that internal deposition of shell material to reinforce the vulnerable apical part of the shell was responsible for the observed internal narrowing of the internal chamber. The flattening of the thickened parts of the shell is likely a consequence of the pervasive decalcification of calcareous shells in the Chengjiang biota, although it is also possible that the internal deposits were poorly mineralized in the first place. In either case, it is clear that *P. diania* did not have a pedicle or any other apical soft parts extending through the shell. This is supported by the lack of any evidence, in carbonate or shale-hosted hyolith specimens, regarding an apical opening that would connect the proposed pedicle (including the reported coelomic cavity) with the soft tissues inside the conch of the hyolith [2].

## Discussion

### Hyolith life modes

**Figure 8.**
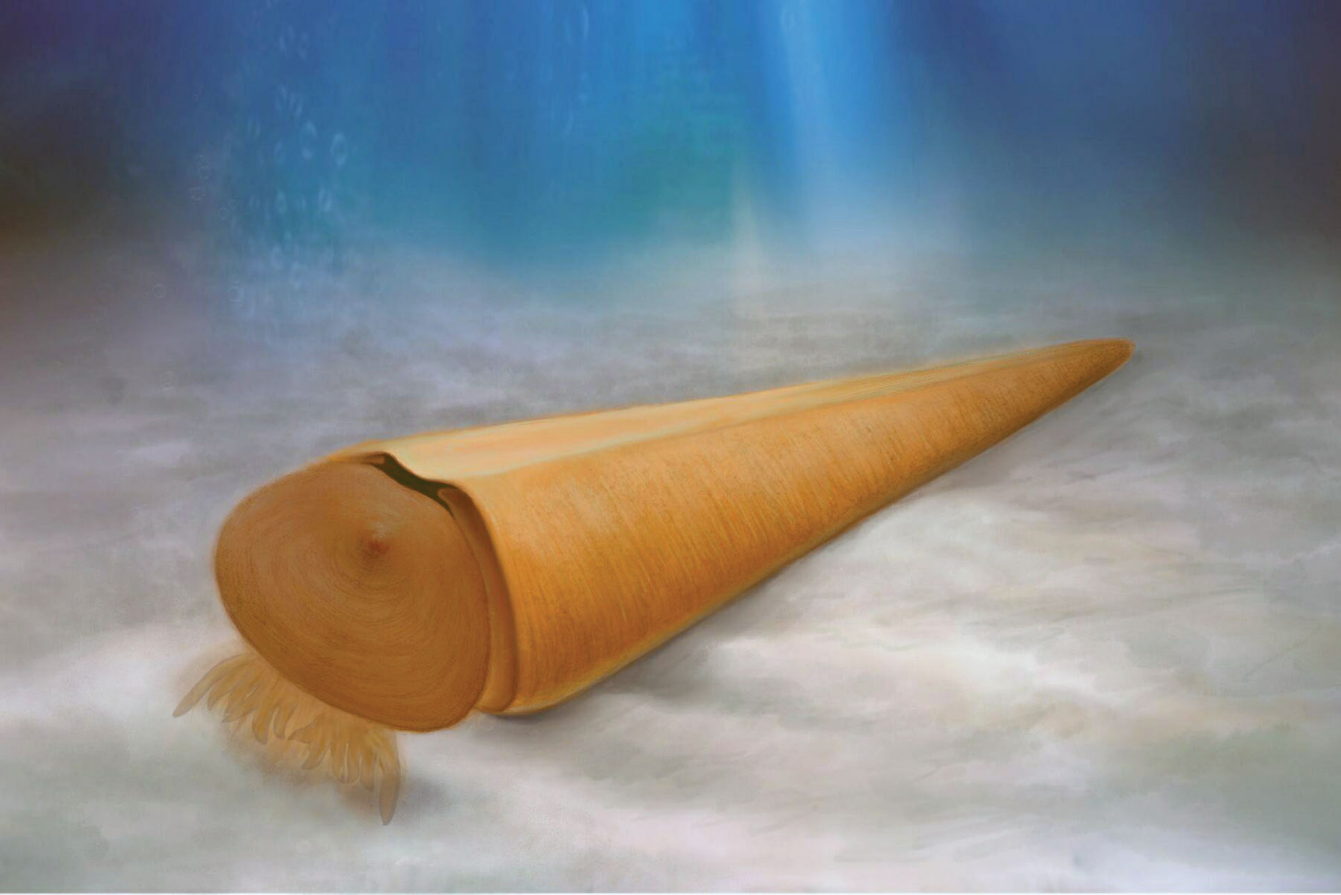
Reconstruction of *Triplicatella opimus* from the Chengjiang Lagerstätte in a proposed deposit-feeding lifestyle.

It has been suggested by many authors [5–7,36,37] that the helens of hyolithids served to lift the apertural end of the conch above the seafloor to avoid fouling the feeding organ. However, orthothecids significantly lack helens and a ventral ligula on the conch aperture. Instead, the planar operculum was retractable inside the conch and the hyolithid mode of life as most recently reconstructed by Moysuik *et al.* (Fig. 3g in [7]) cannot be extended to orthothecids. This observation corresponds to morphological differences in the tentaculate feeding organ of the orthothecid *Triplicatella* documented here with the more tuft-like arrangement of tentacles in *Triplicatella* (Fig. 8), suggesting that *Triplicatella* used its tentacular food-gathering organ in a different way than *Haplophrentis* did.

The triangular cross-section of the conch with a broad, flattened ventral surface, together with the lack of helens, suggests that *Triplicatella* reclined on the seafloor, resting on the ventral surface of the conch. The dorsal attachment of the operculum in *Triplicatella* shows that the principal opening of the shell was along the ventral surface, close to the sediment–water interface and the arrangement of the feeding organ would have been with the tentacles extending towards the sea floor (Fig. 8). This life position does not appear optimal for filtration and, although some other suspension-feeding taxa, such as strophomenid brachiopods, do live at the sediment–water interface, their feeding organs are always orientated into the water column [38], not towards the sediment as in *Triplicatella*. Despite the imperfect preservation of the tentaculate feeding organ of *Triplicatella*, the anterolateral arms, which are generally preserved, only extend by approximately 50% of the width of the operculum, unlike the gullwing-shaped bandof *Haplophrentis* that can extend the full width of the operculum (Fig. 2 in [7]). This suggests that the tuft-like feeding apparatus of *Triplicatella* consisted of a much smaller surface area when compared to the feeding apparatus of *Haplophrentis* (Fig. 2 in [7]), providing little support that this structure in *Triplicatella* would have been efficient for suspension feeding. Instead, we propose that the tentaculate organ in *Triplicatella* was used for collecting food particles directly from the sediment. This general mode of life has been suggested for orthothecids previously [6,39–41] based on the possession of a spiral gut (hyoliths conversely possess a tubular U-shaped gut) that had been argued as an adaptation to deposit feeding, as the increased surface area of the gut would promote the absorption of nutrients mixed with ingested sedimentary particles. The soft parts of *Triplicatella* presented herein provide further evidence to support this claim.

A reclining mode of life was proposed by Sun *et al.* [9] for *P. diania*. As discussed above, we find no evidence that this, or any other hyolith, anchored its conch to the sediment via a pedicle with a basal attachment disc as proposed by Sun *et al.* [9]. The apical end of the conch was closed throughout ontogeny and all hyoliths were either free lying or sediment stickers [42] on the surface of the seafloor. Despite this life position, Sun *et al.* [9] interpreted *P*. *diania* to have been a suspension feeder. However, it is hard to reconcile this life mode (ventral surface lying directly on the sediment) with a suspension-feeding habit, as the tentacular feeding organ of the orthothecid in this life position is aimed directly towards the sediment on the seafloor (Fig. 3 in [9])—a less than ideal arrangement for filtering particles from the water column. Unlike hyolithids that have been interpreted, by means of their helens, as having the ability to actively move over the seafloor (although to what extent remains to be seen) [5,43], there is no evidence of helens or even muscle scars in orthothecids and it is likely that they remained stationary on the seafloor. The inability to move does not necessarily contradict our deposit-feeding interpretation, as there are several species of pelecypods, for example, in the modern world that are stationary surface-deposit feeders [44].

### Implications for lophotrochozoan evolution

The relationship of hyoliths to modern animal groups has been extensively debated in the past [1], with most authors proposing an affinity with either the Mollusca [45] based mainly on similarities in shell morphology and structure or the Sipuncula [46] based on the spiral, U-shaped gut. The unique combination of characters such as helens and a complex muscular system has also prompted others to argue that hyoliths represent an ‘extinct phylum’ [47] or simply a trochozoan of uncertain affinity [14]. However, it was the recent discovery of hyoliths with an extendable, tentacle-bearing feeding organ, interpreted as a lophophore, that saw the group placed within the lophophorates, closely related to the Brachiopoda and Phoronida [7]. A recent study has further emphasized this relationship, placing hyolithids within the Brachiopoda crown group [8]. This notion also received apparent support by the reported presence of a brachiopod-like pedicle in an orthothecid hyolith, although hyoliths were now interpreted as a sister group to the brachiopod crown group [9]. According to these interpretations, both hyoliths and brachiopods were derived from phosphatic-shelled tommotiids with tubular multi-element scleritomes and vermiform, sclerite-bearing organisms such as *Halkieria* [9,48–50].

The soft-part morphology of the orthothecid *T. opimus* from the Chengjiang biota confirms the presence of a tentaculate feeding organ in orthothecids, demonstrating that both recognized orders of hyoliths possessed a tentaculate feeding organ. The differences in body plan and life mode between *Triplicatella* and *Haplophrentis* have implications for the interpreted relationship between hyoliths and lophophorates. In the fossil record, orthothecid hyoliths appear before the first hyolithids by a considerable margin [51], with the first orthothecids occurring in the middle of the Fortunian Stage [52,53] while the first hyolithids probably appeared some time toward the end of Stage 2. Although a number of early hyolith taxa have been referred to the hyolithida based only on a sub-triangular cross-section of the conch (compare the previous assignment of *T. opimus* to the hyolithida), the first hyoliths where evidence for the presence of helens can be observed are *Parkula bounites* and *Parakorilithes mammilatus* (*Hyptiotheca karaculum sensu* Bengtson *et al.* 1990 [15,54]) from the *Micrina etheredgei* zone of South Australia (C.B.S., personal observation), which is equivalent to the upper part of Cambrian Stage 2 [52,55]. Assuming that the interpreted life mode of *Haplophrentis* (where the hyolith depends on the helens to lift the apertural end of the conch above the sediment–water interface [5–7]) is correct, filter feeding in hyoliths may have evolved with the appearance of helens. Hyolithids probably evolved from an orthothecid ancestor lacking helens and, consequently, orthothecids appear to be paraphyletic. The non-filter-feeding apparatus of *Triplicatella* documented here (Fig. 8) may represent the ancestral condition in hyoliths with filter feeding in hyolithids as a secondary adaptation.

Food-collecting tentacles have evolved multiple times among lophotrochozoans and, in addition to the Lophophorata, morphological similarities can be easily drawn between hyolith tentacles and the captacula of modern scaphopods and tentacles of sipunculans. Hyoliths have been directly compared to both groups [45,46] and indeed the function of these tentacular structures may have been comparable. However, the distribution of the tentacles along two lateral bands or arms in *Haplophrentis* and *Triplicatella* is clearly different from the circum-oral distribution of tentacles in scaphopods and sipunculans, and the structures are probably not homologous. Sipunculans are currently considered to be nested within or close to the Annelida [56] and such a phylogenetic position would be difficult to reconcile if the tentacular structures of hyoliths and sipunculans were considered homologous. Coelomate worms have been documented from Cambrian Stage 3 that exhibit morphological features considered typical of sipunculans, such as an anteriorly tapering body that may be wrinkled or covered in fine papillae, possession of a caudal appendage and a retractable introvert armed with hooks [57]. Hyoliths lack these distinctive characters and the differences in body plan [58] together with the possession of a mineralized exoskeleton provides little support for a sipunculan affinity of the Hyolitha.

Despite being frequently compared to molluscs, hyoliths conspicuously lack molluscan apomorphies, such as a foot or radula [6,7], and, without one of these key morphological features, a close relationship to the Mollusca is difficult to justify. The proposed close relationship of hyoliths to molluscs has been predominantly based on similarities in shell structure, where the lamellar–fibrillar microstructure of the hyolith conchs is reminiscent of microstructures present in a range of Cambrian molluscs [13]. However, many other Cambrian shelly fossils, such as hyolithelminths and possibly anabaritids, also possess fibrous microstructures. As such, a fibrous shell structure alone does not conclusively support any particular phylogenetic position for the Hyolitha [13]. More recently, Li *et al.* [12] documented foliated lamellar shell structures in both hyolithids and orthothecids that are extremely similar to the shell structures of coeval molluscs [11,12], suggesting homology between the shell of hyoliths and early molluscs (helcionelloids and bivalves; [12]). A mollusc-like periostracum has also recently been demonstrated in hyoliths [13]. The significance of these shared shell structures amongst Cambrian shelly fossils is currently unclear, but the possibility that such features represent ancestral characteristics that extend back to the stem of the Lophotrochozoa is intriguing.

The presence of a true lophophore and a brachiopod-like fleshy pedicle for anchoring the conch in soft sediment would have been strong evidence that hyoliths were indeed closely related to, or even falling with, the Brachiopoda [9]. However, as shown here, the pedicle in the supposed pedunculate hyolith from the Chengjiang biota (*P. diania*; [9]) in reality represents a partly crushed apical shell and not an anchoring organ, and the feeding apparatus of *Haplophrentis* bears few morphological similarities with lophophores from coeval (and older) brachiopod taxa. The absence of a pedicle and a lophophore prevents hyoliths from being strictly assigned to the Lophophorata and, as such, the close relationship with crown-group brachiopods [7–9] is not supported here. The recently reinterpreted stem-group brachiopod *Yuganotheca* possesses a definitive functional pedicle, a horseshoe-shaped tentacular apparatus and a brachiopod-like bivalved shell with pinnate mantle canals and marginal chaetae [26], characteristics that do merit placement in the lophophorates and potentially as a stem-group brachiopod [9].

Hyoliths, however, conspicuously lack these morphological characters, distancing the group from a close relationship with the Brachiopoda. In the phylogenetic tree presented by Sun *et al.* [9], a sister-group relationship between hyoliths and crown-group brachiopods was supported by the possession of a bivalved shell enclosing a filtration chamber and the differentiation of cardinal areas in the dorsal and ventral valves. We have herein questioned the filtration ability of orthothecids and the identical coding of the ‘cardinal area’ in hyoliths and brachiopods is done under the large assumption that these structures are homologous [9]. Considering that the shell structures of hyoliths and brachiopods bear few similarities (rather greater similarities exist with molluscan shells), homology of these structures appears dubious. Sun *et al.* [9] provide no reason for this speculative homology, only stating that ‘these variable characteristics are easily reconciled with a brachiopod affinity’, and continue by listing brachiopod traits such as possessing a pedicle and lophophore to support their claim of a brachiopod relationship. The questionable nature of these characters has not provided clarification, but only served to muddy the waters regarding the precise phylogenetic affinity of hyoliths. The exact relationship between the lophotrochozoan phyla is still uncertain [16,59,60] and the highly disparate morphology of the earliest probable fossil lophotrochozoans (i.e. halkieriids, tommotiids, hyoliths and helcionelloid molluscs) further complicates the picture.

We consider that the possession of this tentaculate feeding organ (that is not a lophophore) in combination with the molluscan shell structure suggests that hyoliths occupied a more basal position in the Lophotrochozoa, rather than a sister group to the brachiopod crown group [9] or within the brachiopod crown group [8] in the Lophophorata. This is also in accordance with the very early (Fortunian) appearance of hyoliths in the fossil record, well before the first appearance of the first conclusive lophophorate group members (i.e. tommotiids, brachiopods; [61]). If our interpretation of the feeding apparatus of *Triplicatella* is correct, then feeding directly from the sediment may have been the ancestral condition in the Hyolitha, with filter feeding emerging with the evolution of helens. That said, we note that *Triplicatella* and *Haplophrentis* are currently the only two genera of hyoliths that have been documented with the feeding apparatus preserved. It has been suggested previously [6] that hyoliths may have had a relatively generalized feeding organ that could adapt to various feeding strategies depending on the situation (presumably environmental conditions). The broad similarities in the structure of the feeding organs of *Haplophrentis* and *Triplicatella* could provide some support for this hypothesis, although further study and specimens are needed for a reasonable appraisal.

## CONCLUSIONS

We conclude that, presently, no credible evidence exists to suggest that hyoliths belong within the lophophorates, sharing a close phylogenetic relationship with the Brachiopoda. The tentaculate feeding organ of hyoliths [7] is not a lophophore and may have been originally adapted to deposit feeding, with filter feeding evolving later in hyolithids with the appearance of helens. Further, the purported pedicle in orthothecid hyoliths from Chengjiang [9] represents a partly crushed apical shell section and is not a biological analogue to the complex organ that constitutes a brachiopod pedicle. Together with new data on hyolith shell ultrastructures [11–13], the re-evaluated evidence is more parsimonious with hyoliths as a basal lophotrochozoan rather than having a lophophorate ffinity.

## Supplementary Material

nwz161_Supplemental_File

## DATA ACCESSIBILITY

All the specimens dealt with in this paper are deposited in the Early Life Institute and Department of Geology (http://geology.nwu.edu.cn/). Correspondence and requests for materials should be addressed to Z.Z.F. (elizf@nwu.edu.cn).
