# Supplementary material for: Are hyoliths Palaeozoic lophophorates?
Source: Natl Sci Rev. 2019 Oct 25;7(2):453–69. doi: 10.1093/nsr/nwz161 (PMC8289160; doi:10.1093/nsr/nwz161)
Supplement: nwz161_Supplemental_File [file nwz161_supplemental_file.docx]

*Supplementary material* (SM) for

**Are hyoliths Palaeozoic lophophorates?**

**F. Liu, C.B. Skovsted, T.P., Topper, Z-F, Zhang and D-G, Shu**

1. **Material and Methods**

**1.1 *Triplicatella opimus* from the Chengjiang biota**

Approximately 446 specimens of hyoliths have been collected from 8 localities of the Chengjiang Lagerstätte distributed on both sides of Dianchi Lake of Kunming, eastern Yunnan by the working team of the Early Life Institute of Northwest University (Prefix: ELI). All these collections were approved by the Ministry of Land and Resources of China, and no particular licences are required for accessing these fossils deposited in the Early Life Institute (ELI), Northwest University, Xian. The majority of samples examined here were derived from sections at Haikou and Erjie towns on the west bank of Dianchi Lake, where the hyoliths exhibit exceptional preservation of soft tissues. The stratigraphy and fossil localities have been previously documented in detail [1]. In our collection, there are 97 specimens that can be definitely referred to *Triplicatella opimus*. In the samples of *T. opimus*, 17 specimens can be discerned preserved with imprints of tentacular feeding organs (Figs. 1A,D-H and 3E,I ). Nearly all the opercula examined in this study are preserved as internal moulds or moulds with some preserved remains of muscles or other soft tissues or organs (Figs. 1,2,3).

The Chengjiang fauna is recovered from the Yu’anshan Member (*Eoredlichia* Zone) of the upper part of the Early Cambrian Heilinpu (formerly Qiongzhusi) Formation [2]. However, some recent authors considered the Yu’anshan Member as a separate Formation (e.g. [3] and references therein), deposited during Unnamed Cambrian Stage 3 (approximately equivalent to the Atdabanian Stage of Siberia). Numerically, hyoliths are one of the most abundant skeletonized fossils from the Yu’anshan (Chengjiang) deposits. Most specimens in our collection are revealed by means of splitting the mudstone matrix along bedding planes so as to reveal casts or internal moulds of conchs (Figs. 1A and 2), in some cases preserved with their respective opercula (Figs. 1A and 2). They can be preserved as separate individuals or populous occurrence on a single slab of mudrock (Fig. 2D).

The specimens usually retain a thin coat of clay matrix inside the conchs (Figs. 1A and 2B,C). Preserved soft parts associated with the opercula include the lateral edges of the shell secreting tissue and the muscular attachment of the conch and operculum (Figs. 1A and 2E). Most importantly, at least 17 specimens show imprints of a tentaculate feeding organ extended outside (Fig. 1A, D-G) or retracted (Figs. 1H and 3E, I) backwards along the ventral margins of the operculum.

- 1. **The hyoliths from the Shipai Formation**

About 380 shale-hosted macroscopic specimens and abundant small shelly fossils of hyoliths have been collected from the Shipai Formation near Xiachazhuang Village, Zigui county of western Hubei Province, South China. The absolute age of Shipai Formation is unclear [4], but this formation is typically correlated with Cambrian Stage 4 [5]. The Shipai Formation is mainly composed of yellow-green muddy-shales and siltstone and thin calcareous interbeds, yielding abundant fossils including brachiopods, hyoliths, arthropods, palaeoscolecids (Cycloneuralia), sponges, chancelloriids and some problematic organisms [6].

Thousands of small shelly fossils (including many hyoliths) were retrieved through acetic acid (~10%) maceration of two calcareous pelite layers interbedded in the green-yellowish shale. Hyoliths were handpicked out from the residues and selected specimens were coated with gold and studied using a scanning electron microscope (SEM) at the State Key Laboratory of Continental Dynamics, Northwest University.

- 1. **Methods**

Cracked-out fossils of hyoliths were examined under a binocular Zeiss Zoom Stereomicroscope and photographed dry with common external light by a stereophotographic Zeiss Smart Zoom 5, using different illuminations for particular views when high contrast images were required. Some specimens were also photographed using the Fluorescence Stereomicrograph system Nikon SMZ1500 at the Department of Geology, Northwest University. To understand the preservation of the fossils and their chemical composition, selected specimens were also examined by non-destructive Micro X-ray Fluorescence (μ-XRF). Some uncoated specimens were analysed by a FEI Quanta 650 scanning electron microscope (backscattered- a Backscatter Scanning Electron Microscope (BSEM); environmental mode-Energy Dispersive X-ray spectrometry (EDS) system) to examine microstructures of fossils and taphonomic features, at the State Key Laboratory of Continental Dynamics, Northwest University, Xi’an.

**2. Description and comparison of hyoliths from Chengjiang**

At least four species of hyoliths have been described in general descriptions of the Chengjiang biota [7-9] but these have never been systematically described in detail and are in need of taxonomic revision. As mentioned in the text, our material was most recently informally described under the name *Linevitus opimus* Yu, 1974 [8], however the lack of cardinal processes and clavicles in the operculum indicates that the designation to this hyolithid is incorrect. Rather, we consider this taxon to be most closely comparable to the orthothecid genus *Triplicatella,* which was reported from small shelly fossil collections as associated conchs and opercula by Skovsted et al [10]*.* The operculum of *Triplicatella opimus* from the Chengjiang Lagerstätte is nearly flat, sub-triangular or heart-shaped, matching the apertural outline of the conch (Figs. 1A and 2). It shows no evidence of cardinal processes or clavicles on the internal surface (unlike other hyolith opercula in the Chengjiang Biota). The lateral margins of the operculum are adorned with wide folds (Figs. 1A, G, H and 2B, D, E) and two shorter, divergent dorsal folds are present on the dorsal margin while the ventral margin has a wide, weakly expressed median fold (Figs 2E and 3A, E, I). *Triplicatella opimus* from the Chengjiang Lagerstätte has a rapidly and evenly expanding conch with a sub-triangular cross section (Figs. 1A and 2). The dorsum bears a deep carina separated by two crests (Figs 1A and 2B,C). The external ornament is composed of fine growth lines. The conch is strongly mineralised and all specimens show signs of brittle deformation (Fig.2), although most specimens retain substantial topography and are partly filled with sediment (Figs. 1A and 2B, C). The apical portion of the conch is often missing after breaking along simple transverse septa sealing off the apex. After comparison of the shape and morphology of the operculum , the operculum here described from Chengjiang is closely comparable to the operculum of *Triplicatella disdoma* from South Australia and *Triplicatella peltata* from Greenland that also lack clavicles and cardinal processes [10-12]. However, the Chengjiang species differs in the sub-triangular outline of the operculum and the less pronounced ventral folds of the operculum and as a result, represents a separate species of *Triplicatella*.

**References**

1. Zhang ZF, Li G and Emig CC et al. Architecture and function of the lophophore in the problematic brachiopod *Heliomedusa orienta* (Early Cambrian, South China). *Geobios* 2009; **42**, 649-661.(doi:10.1016/j.geobios.2009.04.001)
2. Luo HL, Hu SX and Chen LZ et al. *Early Cambrian Chengjiang Fauna From Kunming Region, China* 1999.Yunnan Science and Technology Press, Kunming, China.1-129. (In Chinese with English summary)
3. Zhao FC, Zhu MY and Hu SX. Community structure and composition of the Cambrian Chengjiang biota. *Science China Earth Science* 2010; **53**, 1784-1799.(doi:10.1007/s11430-010-4087-8)
4. Zhang XL and Hua H. Soft-bodied fossils from the Shipai Formation, lower Cambrian of the Three Gorges area, South China. *Geological Magazine* 2005; **142**(6), 699—709.
5. Zhang ZF, Zhang ZL and Holmer LE et al. First report of linguloid brachiopods with soft parts from the lower Cambrian (Series 2, Stage 4) of the Three Gorges area, South China. *Annales De Paléontologie* 2015; **101**(3), 167—177.
6. Liu F, Chen FY and Chen YL et al. Note on the Shipai biota from the lower Cambrian (Series 2 ,Stage 4) of the Three Gorges Area, South China. *Acta Palaeontologica Sinica* 2017; **56**(4), 516-528. (in Chinese with English abstract)
7. Hou XG, Bergström J and Wang HF et al. *The Chengjiang Fauna: Exceptionally well-preserved animals from 530 million years ago* 1999; Yunnan Science and Technology Press, Kunming.1-80. (In Chinese with English summary)
8. Hou XG, Siveter DJ and Siveter DJ et al. *The Cambrian Fossils of Chengjiang,China, the flowerring of early animal life* 2017; John Wiley & Sons, Ltd.102-113.(doi:10.1002/9781118896372.ch16)
9. Chen JY. *The dawn of animal world* 2004; Jiangsu Science and Technology Press, Nanjing, China.1-366. (in Chinese with English summary).
10. Skovsted CB, Topper TP, and Betts MJ et al. Associated conchs and opercula of *Triplicatella disdoma* (Hyolitha) from the early Cambrian of South Australia. *Alcheringa: An Australasian Journal of Palaeontology* 2014; **38**(1):6.
11. Bengtson S, Morris SC and Cooper BJ et al. Early Cambrian shelly fossils from South Australia. *Memoirs of the Association of Australasian Palaeontologists* 1990; **9**, 211-231.
12. Skovsted CB, Peel JS and Atkins CJ. The problematic fossil *Triplicatella* from the Early Cambrian of Greenland, Canada, and Siberia. *Canadian Journal of Earth Sciences* 2004; **41**, 1273-1283.(doi:10.1139/e04-066)
